# Epidemiology of *bla*_CTX-M_-Positive *Salmonella* Typhimurium From Diarrhoeal Outpatients in Guangdong, China, 2010–2017

**DOI:** 10.3389/fmicb.2022.865254

**Published:** 2022-06-17

**Authors:** Qi Jiang, Bi-xia Ke, De-shu Wu, Dong Wang, Liang-xing Fang, Ruan-yang Sun, Min-ge Wang, Jing-er Lei, Zheng Shao, Xiao-ping Liao

**Affiliations:** ^1^National Risk Assessment Laboratory for Antimicrobial Resistance of Animal Original Bacteria, South China Agricultural University, Guangzhou, China; ^2^Laboratory of Veterinary Pharmacology, College of Veterinary Medicine, South China Agricultural University, Guangzhou, China; ^3^Guangdong Provincial Center for Disease Control and Prevention, Guangzhou, China; ^4^Guangdong Laboratory for Lingnan Modern Agriculture, Guangzhou, China

**Keywords:** *Salmonella* Typhimurium, *bla*_CTX-M_, diarrhoeal outpatients, Guangdong, bacterial persistence

## Abstract

*Salmonella enterica* can lead to intestinal diarrhea, and the emergence and spread of cephalosporin-resistant *Salmonella* have brought great challenges to clinical treatment. Therefore, this study investigated the prevalence and transmission of *bla*_CTX-M_ genes among *S.* Typhimurium from diarrhoeal outpatients in Guangdong, China, from 2010 to 2017. A total of 221 *bla*_CTX-M_-positive isolates were recovered from 1,263 *S.* Typhimurium isolates from the facal samples of diarrhoea patients in 45 general hospitals from 11 cities. The most popular CTX-M gene was *bla*_CTX-M-55_ (39.6%, 72/182) in the CTX-M-1 group, followed by *bla*_CTX-M-14_ (22.5%, 41/182) and *bla*_CTX-M-65_ (19.2%, 35/182) in the CTX-M-9 group. The isolates that carried *bla*_CTX-M-9G_ had significantly higher resistance rates to multiple antibacterials compared with *bla*_CTX-M-1G_ (*p* < 0.01). Meanwhile, PFGE analysis not only showed the clonal transmission of *bla*_CTX-M-55/14/65_-positve isolates of diarrhoeal outpatients’ origins from different hospitals in Guangdong province, but also the characteristic of *bla*_CTX-M-55/14/65_-positve isolates’ bacterial persistence. Multilocus sequence typing (MLST) analysis indicated that these *S.* Typhimurium isolates possessed ST34 and ST19. Furthermore, genomic Beast phylogenomic analysis provided the evidence of a close relationship of *bla*_CTX-M_-positive *S.* Typhimurium isolates between the outpatients and pork. Most *bla*_CTX-M-55/14/65_ genes were transmitted by non-typeable or IncI1/IncFII/IncHI2 plasmids with the size of ranging from ~80 to ~280 kb. Moreover, whole-genome sequencing (WGS) analysis further revealed that *bla*_CTX-M-55/14/65_ coexisted with other 25 types of ARGs, of which 11 ARGs were highly prevalent with the detection rates >50%, and it first reported the emergence of *bla*_TEM-141_ in *S.* Typhimurium. This study underscores the importance of surveillance for *bla*_CTX-M_-positive microbes in diarrhea patients.

## Introduction

*Salmonella enterica* is a zoonotic pathogen of substantial concern to human and animal health (Yin and Zhou, 2018). What’s more, it is a leading cause of morbidity and mortality in people worldwide, with approximately 90 million cases of gastroenteritis and 150,000 associated deaths (Xu et al., 2021). So far, more than 2,610 *Salmonella* serovars have been identified, while salmonellosis is caused mainly by *S. enterica* serovars Typhimurium, Enteritidis and Dublin (Shi, 2015; Mohammed et al., 2017). Nontyphoidal *S.* Typhimurium is a dominant factor of human gastroenteritis, and improper handling and digestion of inadequately looked food primarily result in the infection. Invasive complications, including meningitis, sepsis and bacteraemia, are very common in infants, the elderly and immunocompromised patients. The disease of *S.* Typhimurium is usually related to contaminated foods, such as pork and fruits, unpasteurized milk and dairy products, and undercooked eggs (Wegener et al., 2003).

In these potentially life-threatening *S.* Typhimurium cases, the antibiotics of choice are fluoroquinolones and extended-spectrum cephalosporins (Diard and Hardt, 2017). Third-generation cephalosporins (3GCs) are used across the world to threat infections caused by *Salmonella*, and subsequently the emergence of resistance attracts particular attention (Whichard et al., 2007). Multidrug-resistant (MDR) *Salmonella* spp. potentially arising for the selective pressure from sustained antimicrobial exposure are more likely to be the causative agents of invasive disease (Okoro et al., 2015). Moreover, the ESBL-producing strains of *Salmonella* have been reported in many regions in China, including Beijing, Shanghai, Guangdong, and Shandong (Cao C. et al., 2021). Worse, ESBL-producing *S.* Typhimurium have increasingly been detected from food animals, even environmental water and human patients (Fu et al., 2020; Ma et al., 2020). Hence, the number of ESBL-*Salmon* has increased worldwide.

TEM, SHV, and CTX-M were the most prevalent ESBL types. It has commonly been found that ESBL-CTX-M is located on plasmids and considered as the most prevalent type of ESBLs in many European countries (Paterson and Bonomo, 2005). At the same time, there is tremendous diversity of *bla*_CTX-M_ genotypes isolated from food animals and human populations. Usually, among the reported bacteria with *bla*_CTX-M-55_-positive or *bla*_CTX-M-14_-positive or *bla*_CTX-M-65_-positive, most are isolated from food and animal sources (Xiang et al., 2015; Zhang et al., 2015; Nadimpalli et al., 2019). A practice was selected for antibiotic resistant *S. enterica* that can spread to human through contaminated foods. However, this practice is not currently monitored or regulated in Guangdong Province.

Therefore, in this study, ESBL-producing *S.* Typhimurium isolates, mainly from diarrheal patients, isolated from Guangdong province, and collected at the Guangdong Provincial CDC during the period of 2010–2017, were investigated to gain insight into their public health impacts.

## Materials and Methods

### Bacterial Isolates, Detection of ESBL/*pAmpC* Genes, and Antimicrobial Susceptibility Testing

A total of 1263 *S.* Typhimuriums were recovered from facal samples of diarrhoea patients in 45 general hospitals from 11 cities of Guangdong province between 2010 and 2017. These isolates were collected by the Guangdong Provincial Center for Disease Control and Prevention (CDC) in a clinic-based *Salmonella* infection surveillance of outpatients with diarrhea, as described previously (Zhang et al., 2013). All 1263 *S.* Typhimurium isolates were incubated on MacConkey agar plates, containing 4 mg/L cefotaxime. The cefotaxime-resistant *S.* Typhimurium isolates were subjected to screening for CTX-M, CTX-M-1G, CTX-M-9G, CMY-2G, SHV, and DHA genes (Supplementary Table S1; Liu et al., 2007), and *bla*_CTX-M-1G/9G_-positive isolates were further subjected to determine the subtypes of ESBL-encoding genes, as previously reported (Zhao and Hu, 2013). The DNA sequences and deduced amino acid sequences were compared with the reported sequences from GenBank. Antimicrobial susceptibility testing was performed on all the CTX-M-producing isolates by the agar dilution method, except for colistin with the broth dilution method. The following antimicrobials were tested: cefotaxime, ceftriaxone, ceftazidime, ceftiofur, meropenem, ciprofloxacin, nalidixic acid, sulfamethoxazole/trimethoprim, gentamicin, amikacin, florfenicol, fosfomycin, azithromycin, doxycycline, olaquindox, tigecycline, and colistin. The results were interpreted according to the Clinical and Laboratory Standards Institute (CLSI, 2018: M100-S25), and veterinary CLSI (VET01-A4/VET01-S2) guidelines (Humphries et al., 2019), and the resistance breakpoints for colistin were interpreted based on EUCAST (>2 mg/L) criteria, respectively. *Escherichia coli* ATCC25922 was used as the quality control strain.

### Molecular Typing

The genetic relatedness of *bla*_CTX-M_-positive *S.* Typhimurium isolates was analyzed by PFGE with the *Xba*I digestion of genomic DNA (Palhares et al., 2014). PFGE patterns were analyzed using BioNumerics software (Applied Maths, Sint-Martens-Latem, Belgium) with the Dice similarity coefficient, and a cut-off value of 85% of the similarity values was chosen to indicate identical or similar PFGE types.

### WGS and Phylogenetic Analysis

Based on the results of PFGE types and resistance profiles analysis, representative *bla*_CTX-M_-positive *S.* Typhimurium isolates (*n* = 57) were selected and their genomic DNA were subjected to 250-bp paired-end whole-genome sequencing (WGS), which at a depth of 100X, using the Illumina MiSeq system (Illumina, San Diego, CA, United States), using default parameters, followed by assembling the 150 bp paired-end Illumina reads using SPAdes v3.6.2 (Humphries et al., 2019). Multi locus sequence typing (MLST), antibiotic resistance genes (ARGs), and plasmid replicon types were analyzed using the CGE server.^1^ Phylogenetic tree for CTX-M-producing isolates was structured on the basis of the core genome using Harvest version 1.1.2 (Treangen et al., 2014), and the corresponding characteristics of each isolate were visualized using online tool iTOL version 4 (Letunic and Bork, 2019). The population structure of each phylogenetic tree was defined using hierBAPS v6.0 (Cheng et al., 2013).

### Conjugation Assay, Gene Location, and Plasmids Analysis

To test the transferability of *bla*_CTX-M_ genes, conjugation experiment was carried out by the liquid mating-out assay, with the streptomycin-resistant *E. coli* C600 as the recipient. Transconjugants were selected on MacConkey agar plates that were supplemented with cefotaxime (2 mg/L) and streptomycin (1,500 mg/L). Antimicrobial susceptibility testing was conducted on transconjugants and the *bla*_CTX-M_ gene was confirmed by PCR, as described above. PCR-based replicon typing was performed for transconjugants, as previously described (Bankevich et al., 2012). To determine the location of *bla*_CTX-M_, plasmids from the selected transconjugants were linearized using S1 nuclease and subjected to PFGE, followed by Southern blot hybridization using a digoxigenin-labeled probe specific for *bla*_CTX-M-1G/9G_, as previously described (Liu et al., 2007).

### Data Availability

All genome assemblies of the 57 *bla*_CTX-M_-positive strains were deposited in GenBank and are registered under BioProject accession number PRJNA797940 and PRJNA629650.

## Results

### Prevalence of CTX-M Genes

**Figure 1:**
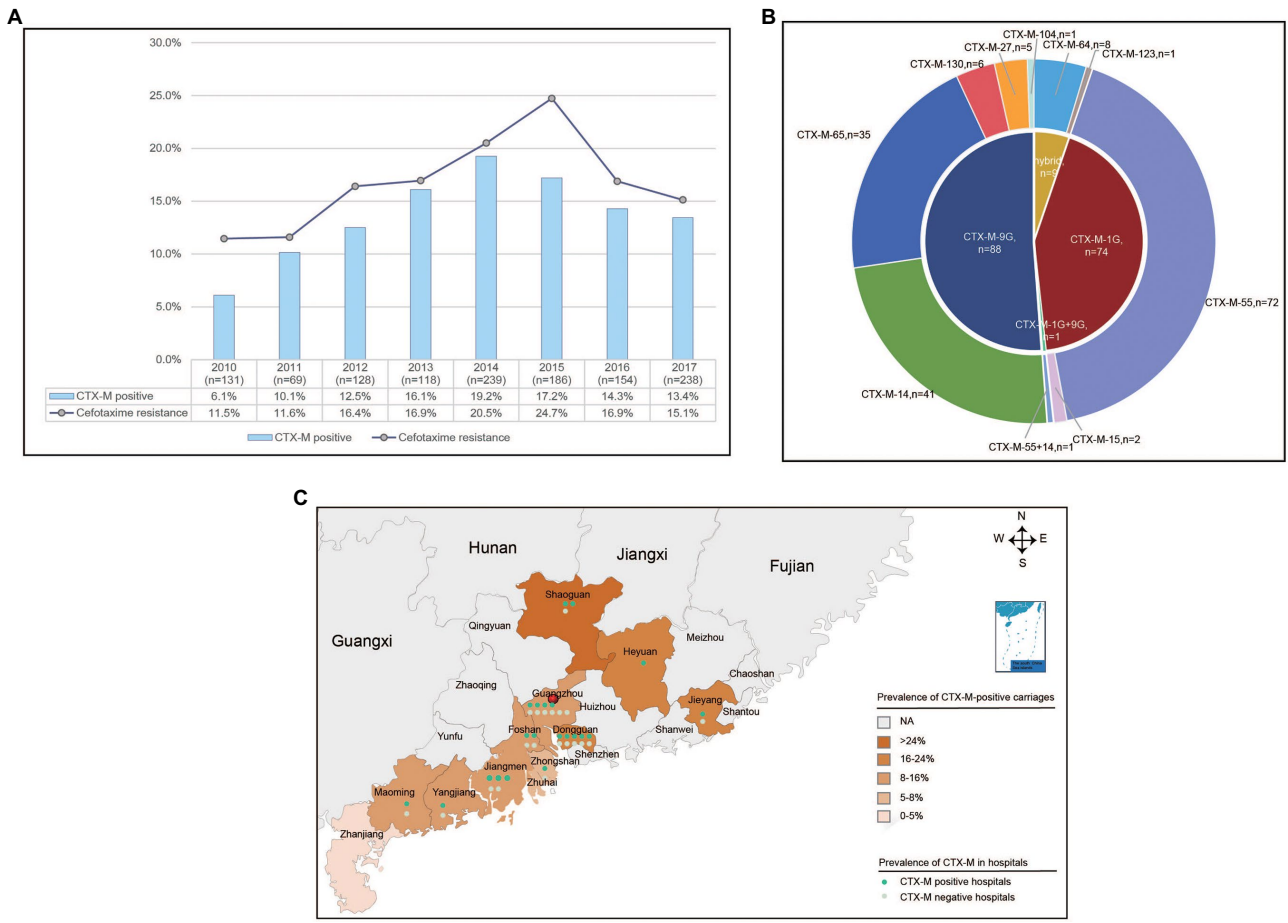
The prevalence of *bla*_CTX-M_-positive *Salmonella* Typhimurium isolates from diarrhoeal patients in 11 hospitals in Guangdong Province, China from 2010 to 2017. **(A)** The rate of resistance to cefotaxime and detection rate of *bla*_CTX-M_-positive *S.* Typhimurium isolates in Guangdong Province from 2010 to 2017. **(B)** The subtypes and numbers of variants in CTX-M. **(C)** The detection rate of *bla*_CTX-M_-positive *S.* Typhimurium isolates in 11 cities in Guangdong Province.

A total of 221 (17.5%) isolates displayed resistance to cefotaxime among the 1,263 *S.* Typhimurium isolates collected in 45 hospitals across 11 cities from Guangdong, China. Of which, 82.4% (182/221) carried one or two *bla*_CTX-M_ variants. In addition, 20.8% (46) isolates contained *bla*_CMY-2G_ gene and 16 (7.2%) isolates harbored *bla*_DHA_ gene, and no *bla*_SHV_ gene was detected among these isolates. A total of nine *bla*_CTX-M_ variants (*bla*_CTX-M-55_, *bla*_CTX-M-14_, *bla*_CTX-M-65_, *bla*_CTX-M-64_, *bla*_CTX-M-130_, *bla*_CTX-M-27_, *bla*_CTX-M-15_, *bla*_CTX-M-104_, and *bla*_CTX-M-123_) were detected in 182 *bla*_CTX-M_-producing isolates, and the most predominant was *bla*_CTX-M-55_ (39.6%, 72/182), followed by *bla*_CTX-M-14_ (22.5%, 41/182) and *bla*_CTX-M-65_ (19.2%, 35/182; Figure 1B). Furthermore, one isolate harbored both *bla*_CTX-M-55_ and *bla*_CTX-M-14_.

The percentages of cefotaxime-resistant isolates and *bla*_CTX-M_-positive isolates had been shifted significantly from 11.5% and 6.1% in 2010 to 24.7% and 17.2% in 2015, but decreased to 15.1% and 13.4% in 2017, respectively (Figure 1A). The *bla*_CTX-M_-positive isolates were identified in 35 hospitals among 11 cities. Of which, Shaoguan had the highest detection power of 25.0%. The mean positive prevalence of *bla*_CTX-M_ carriages was 13.5% among the 12 cities (Figure 1C). Among the patients who were found to be positive for *bla*_CTX-M_-positive *S.* Typhimurium isolates, the median age of patients with *bla*_CTX-M_-producing isolates was 1 year (range 0–90 years), and 90% of patients were <5 years of age. In addition, 70.3% patients were male (Table 1).

**Table 1:** *bla*_CTX-M_-Positive *S.* Typhimurium isolates collected from patient demographics characteristics, Guangdong, 2010–2017 (*N* = 182).

Characteristics	Value
Sex	
M	128
F	53
Unknown	1
Age, y, median (range)	1, (0–90)
Age group, y	
≤5	165
>5	15
Unknown	2

### Antimicrobial Susceptibility

**Figure 2:**
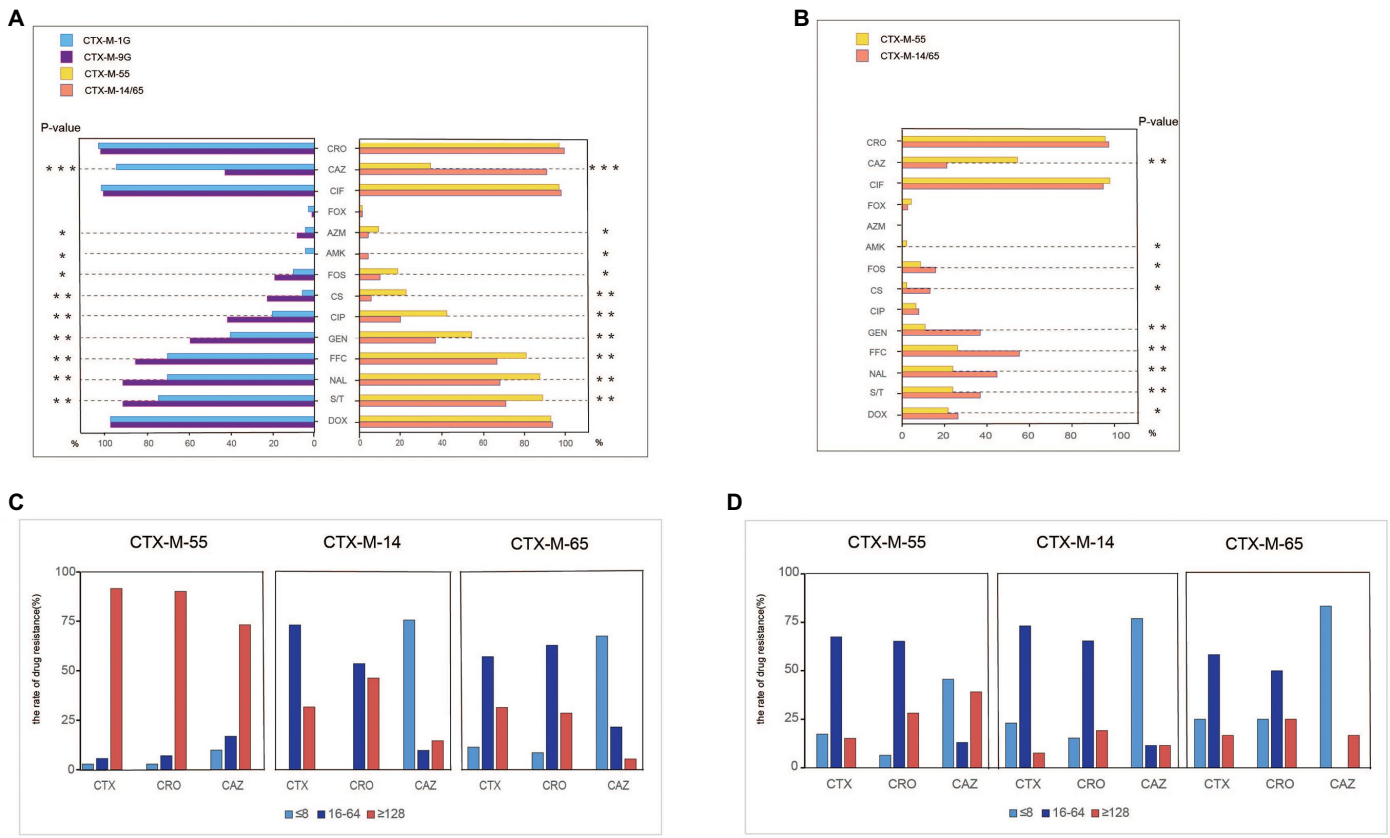
Antimicrobial-resistant phenotypes analysis of *bla*_CTX-M_-positive *S.* Typhimurium isolates and transconjugants isolates. **(A)** Antimicrobial-resistant phenotypes analysis of *bla*_CTX-M-1G/9G/55/14/65_-positive *S.* Typhimurium isolates. **(B)** Antimicrobial-resistant phenotypes analysis of *bla*_CTX-M-1G/9G/55/14/65_-positive *S.* Typhimurium’ transconjugants. **(C)** Anslysis of resistant to CTX, CRO, and CAZ in *bla*_CTX-M-55/14/65_-positive *S.* Typhimurium isolates. **(D)** Anslysis of resistant to CTX, CRO, and CAZ in *bla*_CTX-M-55/14/65_ *S.* Typhimurium’ transconjugants. CTX, cefotaxime; CRO, ceftriaxone; CAZ, ceftazidime; CIF, ceftiofur; FOX, cefoxitin; AZM, azithromycin; AMK, amikacin; FOS, fosfomycin; CS, polymyxin; CIP, ciprofloxacin; GEN, gentamicin; FFC, florfenicol; NAL, nalidixic acid; S/T, sulfamethoxazole/trimethoprium; and DOX, doxycycline. *means statistically different (*p* < 0.05), **means the difference is more significant (*p* < 0.01), ***means the difference is particularly significant (*p* < 0.001).

Antimicrobial susceptibility was tested among the 182 *bla*_CTX-M_-positive *S.* Typhimurium isolates, and most of the isolates showed resistance to sulfamethoxazole/trimethoprim (81.3%), and florfenicol (70.9%), followed by gentamicin (48.4%) and ciprofloxacin (31.3%). Relatively low resistance rates were observed for colistin (14.8%), fosfomycin (14.3%), and amikacin (1.7%). All the 182 isolates were susceptible to meropenem. Of note, the isolates that carried *bla*_CTX-M-9G_ had significantly higher resistance rates to nine antibacterials compared with *bla*_CTX-M-1G_ (*p* < 0.01), including florfenicol, amikacin, gentamicin, ciprofloxacin, nalidixic acid, polymyxin, fosfomycin, azithromycin, and sulfamethoxazole/trimethoprim (Figure 2A). The same scenario was also observed in *bla*_CTX-M-55_, *bla*_CTX-M-14_, and *bla*_CTX-M-65_ positive isolates. However, the isolates that carried *bla*_CTX-M-1G_, including *bla*_CTX-M-55_, had remarkably higher rates of resistance to ceftazidime compared with *bla*_CTX-M-9G_ including *bla*_CTX-M-14_ and *bla*_CTX-M-65_ (*p* < 0.001).

Furthermore, to determine the association between the dominant *bla*_CTX-M_ genes and the 3GCs susceptibility, MICs of cefotaxime, ceftriaxone and ceftazidime were grouped into three levels, namely low resistance level (≤8 mg/ml), medium resistance level (16–64 mg/ml), and high resistance level (≥128 mg/ml; Figure 2C). The majority of *bla*_CTX-M-55_, *bla*_CTX-M-14_, and *bla*_CTX-M-65_-positive *S.* Typhimurium isolates showed moderate and high levels resistance to cefotaxime and ceftriaxone. However, the proportion of high levels of resistance to cefotaxime and ceftriaxone in *bla*_CTX-M-55_-positive *S.* Typhimurium isolates was higher than that of *bla*_CTX-M-14/65_-positive *S.* Typhimurium. It was obvious that most *bla*_CTX-M-55_-positive isolates are resistant to ceftazidime at high levels. In contrast, most isolates *bla*_CTX-M-14_-positive or *bla*_CTX-M-65_-positive were presented low-level resistant to ceftazidime.

### Molecular Typing

The genetic relatedness of *bla*_CTX-M-55_-positive, *bla*_CTX-M-14_-positive and *bla*_CTX-M-65_-positive *S.* Typhimurium isolates were analyzed by PFGE, respectively. PFGE was successfully performed in 71 *bla*_CTX-M-55_-positive isolates and distributed into 26 pulsotypes. The 22 isolates in Type III were obtained in nine hospitals across four cities during 2014–2016. Similarly, the 17 isolates in Type VII were originated in six hospitals from four cities during 2010–2015 (Supplementary Figures S1A, S2). The clonal transmission of *bla*_CTX-M-55_-positive strains was observed at different hospitals in the same city between 2014 and 2016. A total of 21 different pulsotypes were detected among 41 *bla*_CTX-M-14_-positve isolates, and Type I was predominant (*n* = 9, 21.95%; Supplementary Figures S1B, S2). The clonal transmission of *bla*_CTX-M-14_-positive strains was observed at the same hospitals in the same city in 2012. Most importantly, all 35 *bla*_CTX-M-65_-positve isolates were distributed into 15 pulsotypes, and the most predominant Type VIII contained 19 isolates (54.3%) and was originated from nine hospitals in seven cities during 2013–2017 (Supplementary Figures S1C, S4). The spread of *bla*_CTX-M-65_-positve isolates’ clones from Guangzhou and Jieyang was observed.

**Figure 3:**
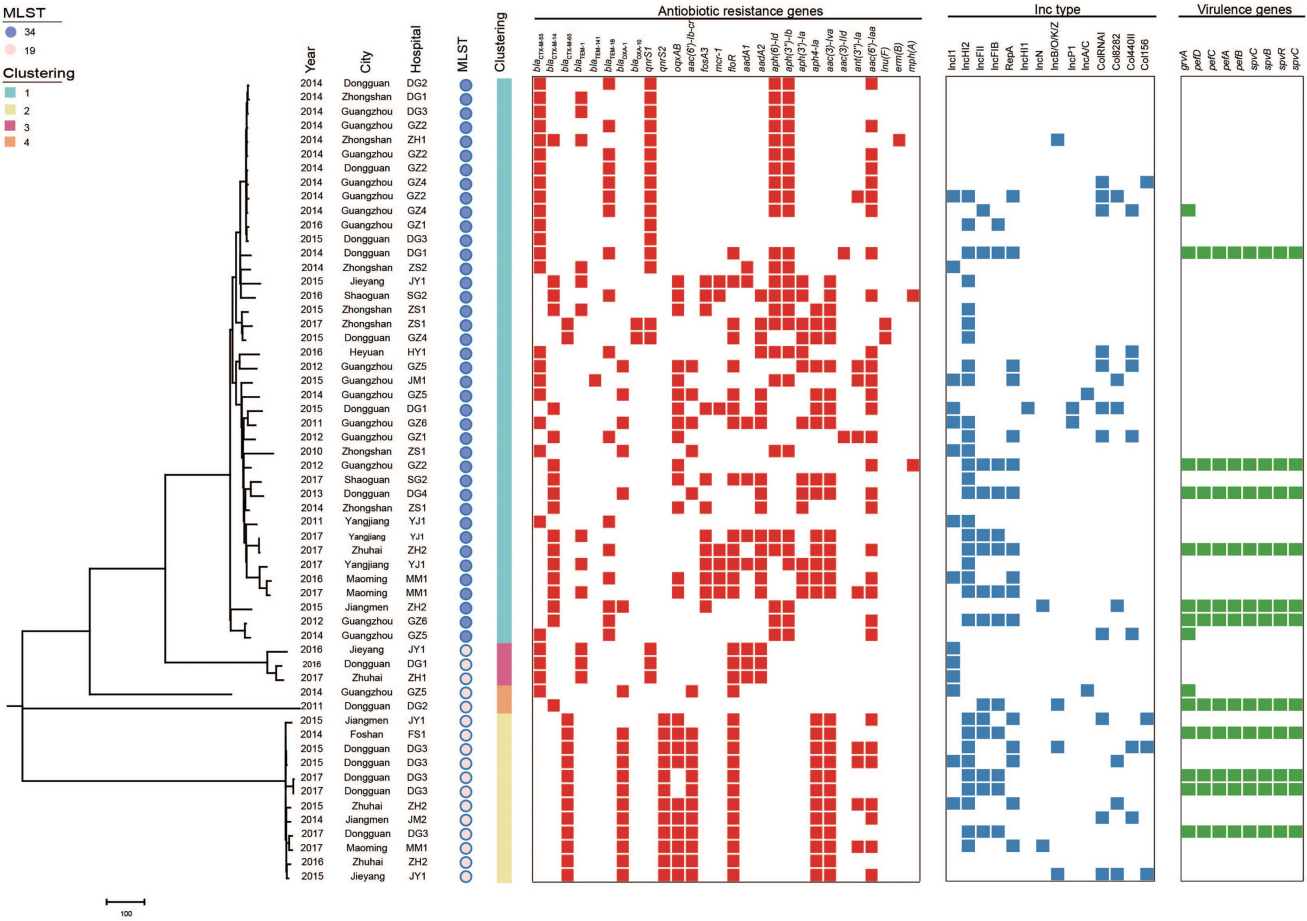
Phylogenetic analysis of *bla*_CTX-M_-positive *S.* Typhimurium isolates in this study (*n* = 57). Bayesian evolutionary tree was constructed using core-genome SNPs. Each isolate is labeled with the city of isolation year, hospital, ST and cluster. The red-filled squares indicate the possession of the indicated antimicrobial resistance genes (ARGs), the blue-filled squares indicate plasmid (Inc type), and the green-filled squares indicate virulence genes.

According to PFGE typing and resistance phenotype, 57 (27 *bla*_CTX-M-55_, 14 *bla*_CTX-M-14_, 15 *bla*_CTX-M-65_, and 1 *bla*_CTX-M-55/14_) *S.* Typhimurium isolates were selected for WGS. *In silico* MLST analysis revealed that these isolates belong to ST34 (*n* = 40) and ST19 (*n* = 17; Figure 3). Among them, most ST34 (3.8%, 15/40) and ST19 (4.1%, 7/17) belong to cluster 1 from Guangzhou and cluster 2/3/4 from Dongguan, respectively.

### Phylogenetic Analysis of *bla*_CTX-M-55/14/65_- Positive *Salmonella* Typhimurium

The population structure was further analyzed by constructing phylogenetic trees based on the core genomes of the 57 *bla*_CTX-M_-positive isolates. Bayesian analysis displayed that all isolates were classified into four different lineages. The major Lineage I belong to ST34 and Lineage II-IV belong to ST19.

**Figure 4:**
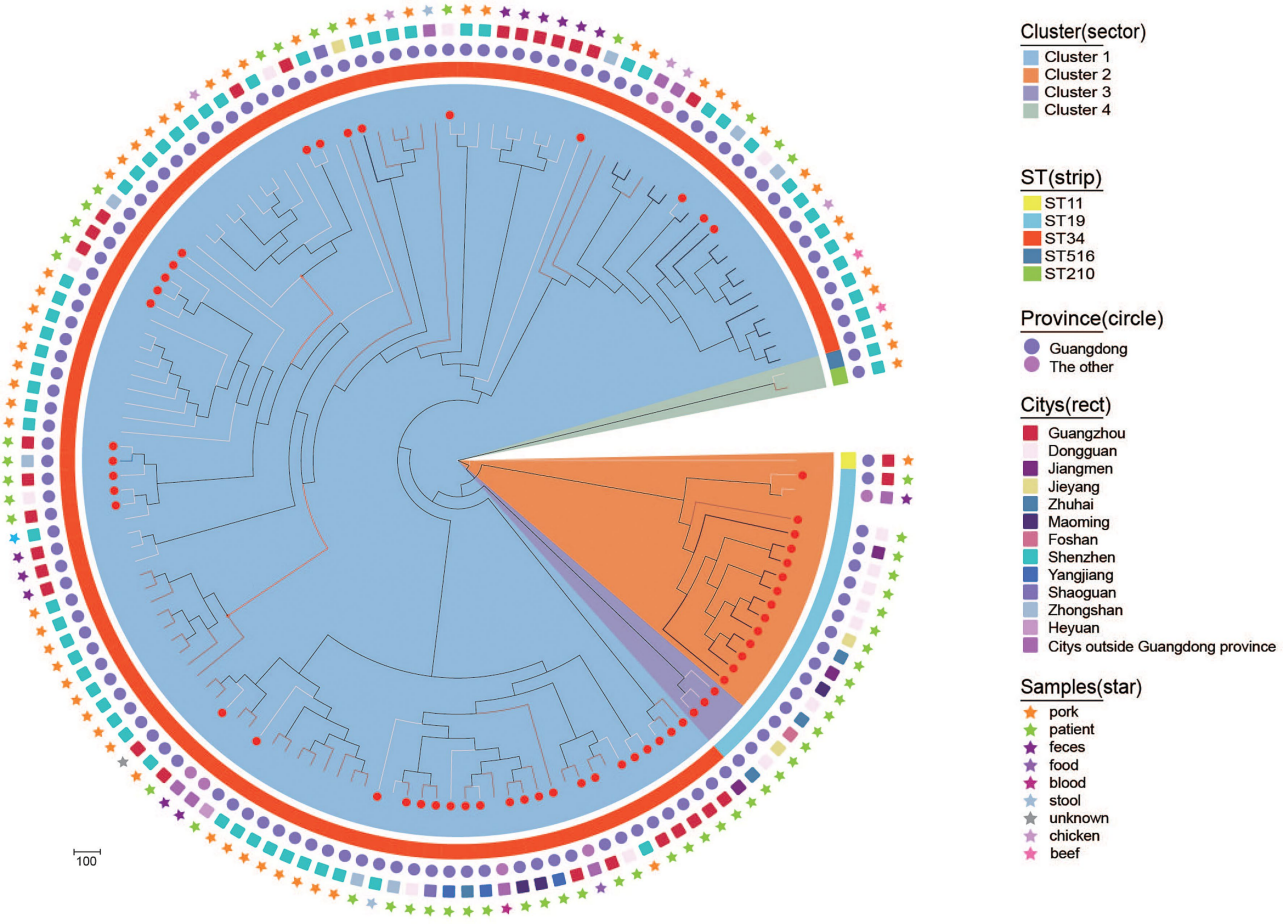
Phylogenetic structures of the *bla*_CTX-M_-positive *S.* Typhimurium isolates from this study and the GenBank database. The Bayesian evolutionary tree shows the relationships among the 141 *bla*_CTX-M-55/14/65_-positive *S.* Typhimurium isolates. Sample source, city, province and ST from which isolates obtained are indicated as star, rect, circle, and strip. *bla*_CTX-M-55_-positive, *bla*_CTX-M-14_-positive, and *bla*_CTX-M-65_-positive *S.* Typhimurium are indicated with a white, black, and red line, respectively. The red dots on the branches represent the bacteria that belong to this study.

To explore the genetic relationships of *bla*_CTX-M-55/14/65_-positive *S.* Typhimurium isolates in this study and other resources in China, 84 *bla*_CTX-M_-positive *S.* Typhimurium isolates (including 36 *bla*_CTX-M-55_, 33 *bla*_CTX-M-14_, and 15 *bla*_CTX-M-65_) were selected from GenBank. A maximum likelihood phylogenetic tree was constructed on the basis of 32,165 core genome single nucleotide polymorphisms (cgSNPs) among 138 isolates (Figure 4). These 138 isolates were mainly distributed in Guangdong province (*n* = 134) and other provinces (*n* = 4), such as Shanghai, Hebei, Jiangxi, and Zhejiang. Notably, these 138 isolates were primarily ST34 and ST19 members and originated from diverse sample types, including humans (patients, synviol fluid and blood culture), food (beef, chicken, feces, pork) and the environments (stool). It should be noted that four *bla*_CTX-M-14_-positive ST34 *S.* Typhimurium isolates from patient samples in three cities, Guangdong (own isolate’ number: 17E74), shared only 64 SNPs with a *bla*_CTX-M-14_-positive ST34 *S.* Typhimurium isolate from a blood culture sample in Jiangxi (accession number SAMN10914546). In addition, a *bla*_CTX-M-55_-positive ST34 *S.* Typhimurium isolate from a patient in Dongguan, Guangdong, in this study (own isolate’ number: L-S2816) shared only six SNPs with a *bla*_CTX-M-55_-positive ST34 *S.* Typhimurium isolates from pork in Shenzhen, Guangdong (accession number SAMN16986615). Finally, one *bla*_CTX-M-65_-positive ST19 *S.* Typhimurium isolate from a patient sample in Zhongshan, Guangdong, in this study (own isolate’ number: 17E594) shared only 28 SNPs with a *bla*_CTX-M-65_-positive ST19 *S.* Typhimurium isolate from pork sample in Shenzhen, Guangdong (accession number SAMN16986937). These data may demonstrate that *bla*_CTX-M_-positive *S.* Typhimurium isolates from human were likely to be closely related to food and environment in China, and the environment and food chain may play an important role in the transmission of *bla*_CTX-M_-positive *S.* Typhimurium isolates.

### Plasmid Analysis

Conjugation experimental results proved that 84 *bla*_CTX-M_-positive plasmids were successfully transferred to *E. coil* C600 recipient strains among 148 *bla*_CTX-M-55/14/65_ positive *S.* Typhimurium isolates. The transconjugants *bla*_CTX-M-55/14/65_-positive mainly showed moderate levels resistance to cefotaxime and ceftriaxone and low levels resistance to ceftazidime. Notably, the proportion of *bla*_CTX-M-55_-positive transconjugants with high levels of resistance to ceftazidime was higher than that of *bla*_CTX-M-14/65_-positive transconjugants (Figure 2B). Meanwhile, the *bla*_CTX-M-55_-positive transconjugants had significantly higher resistance rates to ceftazidime compared with *bla*_CTX-M-14/65_-positive transconjugants (*p* < 0.001; Figure 2B).

In addition to cephalosporins, partial transconjugants displayed resistance to florfenicol (*n* = 33), doxycline (*n* = 29), sulfamethoxazole/trimethoprim (*n* = 28), gentamicin (*n* = 25), fosfomycin (*n* = 17), and azithromycin (*n* = 17). Obviously, the transconjugants that carried *bla*_CTX-M-9G_ had significantly higher resistance rate to seven antibiotics compared with *bla*_CTX-M-1G_ (*p* < 0.01), including amikacin, fosfomycin, gentamicin, polymyxin, florfenicol, sulfamethoxazole/trimethoprim, nalidixic, acid and doxycycline (Figure 2D).

Through conjugation assay and gene location methods, replicon analysis was performed on the *bla*_CTX-M-55/14/65_-positive transconjugants, mainly IncI1(*n* = 49), followed by IncHI2(*n* = 23) and IncFII (*n* = 8; Supplementary Figure S7). Based on PFGE profiles, one isolate from each clonal lineage was selected for *S1*-PFGE and hybridization. For the *bla*_CTX-M-55/14/65_-positive isolates (*n* = 12, 8, and 6, respectively), S1-PFGE and hybridization analyses confirmed that *bla*_CTX-M-55_-positive genes (*n* = 10) from 12 isolates were mainly located on ~76.8 kb plasmids, *bla*_CTX-M-14_-positive genes (*n* = 4) from eight isolates were mainly located on 54.7–80 kb plasmids, and *bla*_CTX-M-65_-positive genes (*n* = 4) were from six isolates mainly located on 216.9–244.4 kb plasmids (Supplementary Figures S5, S6). Primers connecting contigs containing the backbone of different plasmids and *bla*_CTX-M_ genes were used. The results illustrated that IncI1 (65.3%, 32/49), IncFII (50.0%, 4/8) and IncHI2 (30.4%, 7/23) plasmids may be major vectors for the wide dissemination of *bla*_CTX-M-55_, *bla*_CTX-M-14_, and *bla*_CTX-M-65_ genes in *S.* Typhimurium isolates. In addition, WGS analysis revealed that sequenced strains also carry other plasmids, such as IncFIB-type, IncHI1-type, IncN-type and other different kinds of plasmids.

### Resistance Profiles

WGS analysis demonstrated that 57 *bla*_CTX-M_-producing isolates possessed 47 distinct ARGs. Several clinically important ARGs were identified to co-carry with *bla*_CTX-M_, including *mcr-1*, *fosA3*, *oqxAB*, *qnrS1*, *qnrS2*, *aac-(6′)-Ib-cr*, and *floR*, with a prevalence rate from 12.3% to 52.6%. Moreover, *bla*_TEM-141_ (*n* = 1) was first detected in *S.* Typhimurium isolates.

Notably, some ARGs were co-existence with a specific *bla*_CTX-M_ variant. For example, *mcr-1* and *fosA3* were unique to *bla*_CTX-M-14_-positive isolates. *qnrS2*, *aac(6′)-Ib-cr*, and *bla*_OXA-1_ were primarily found in *bla*_CTX-M-65_-positive isolates. In contrast, *qnrS1* and *bla*_TEM-1B_ were largely present in *bla*_CTX-M-55_-positive isolates. Additionally, both *oqxAB* and *floR* mostly presented in *bla*_CTX-M-65_- and *bla*_CTX-M-14_-positive isolates.

## Discussion

In this study, the detection rate of *bla*_CTX-M_-positive *S.* Typhimurium from diarrhoeal outpatients increased from 2010 to 2015 in Guangdong Province, China. It was speculated that *bla*_CTX-M_-positive *S.* Typhimurium outbreaks are linked to the consumption of food animal or raw meat, particularly pork. Firstly, previous studies showed that the swine is one of the major reservoirs for *Salmonella* (Wang et al., 2007; Jackson et al., 2013). Secondly, the data from China’s National Nutrition Survey also displayed that the total pork intake of Chinese residents increased by 73% from 1992 to 2012 (He et al., 2015). It’s worth noting that the overall percentage of cephalosporin use had an upward trend from 2012 to 2017 in hospitals (Branch, 2020), which can give us some hints that the transmission of *bla*_CTX-M_ may be relevant to the selective pressure of cephalosporin antibiotics. Then, it was obvious that the detection rate of cefotaxime-resistant *S.* Typhimurium in Guangdong province steadily decreased from 2016 to 2017. Meanwhile, according to CHINET bacterial resistance monitoring, the detection rate of cefotaxime-resistant *Enterobacter* decreased gradually from 2015 to 2017. Therefore, the long-term monitoring of cephalosporin usage and the prevalence of the *bla*_CTX-M_-positive *Salmonella* are necessary for public health.

In this study, nine *bla*_CTX-M_ variants were detected in 182 *bla*_CTX-M_-producing isolates, and the most predominant was *bla*_CTX-M-55_, followed by *bla*_CTX-M-14_ and *bla*_CTX-M-65_, which is consistent with previous studies (Zhang et al., 2018). Currently, *bla*_CTX-M-55_-positive *Salmonella* has been reported as the dominant genotype in other countries, including Germany, Cambodia, Korea and Vietnam, and was frequently detected in food animals, especially in poultry and pork (Nguyen et al., 2016; Kim et al., 2017; Lay et al., 2021; Pietsch et al., 2021); *bla*_CTX-M-14_-positive *Enterobacteriaceae* has been reported as the dominant genotype in some countries, including China, South Korea, Japan, and Spain, and was frequently detected in food, especially in retail chicken meat and pork (Bai et al., 2016; Shigemura et al., 2020; Wang et al., 2021); *bla*_CTX-M-65_-positive *Salmonella* has been found in China, the United States, and Germany, and was commonly found in food animal sources, especially in chicken (Brown et al., 2018; Martínez-Puchol et al., 2021; Pietsch et al., 2021).

The *bla*_CTX-M-65_-positive *S.* Typhimurium isolates with the same profile were found in the three hospitals (GZ5, JY1, and DG3), which indicated that the clonal dissemination of *bla*_CTX-M-65_-positive *S.* Typhimurium occurred in hospital. Furthermore, a few *bla*_CTX-M-55/14/65_-positive *S.* Typhimurium isolates from 2010 to 2017 showed 100% homology by PFGE analysis, which suggested that the possible long-term outbreaks were caused by clonal transfer of *bla*_CTX-M-55/14/65_-positive *S.* Typhimurium strains within the hospital. WGS demonstrated that these *S.* Typhimurium isolates belonged to ST34 and ST19. In fact, it has been shown that ST34 and ST19 are common *S.* Typhimurium STs responsible for infections worldwide, especially in China (Woh et al., 2021). It has been proved previously that the ST34 *S.* Typhimurium isolates with the highest percentage of MDR are mainly recovered from diarrhea patients (Biswas et al., 2019; Jiu et al., 2020; Luo et al., 2020; Sun et al., 2020). Furthermore, ST19 has been found mostly in human clinical *Salmonella* isolates, but also in animals and the environment, and successful in South African and China. ST19 was only occasionally found in United States and Mexico and coexists with quinolone resistance genes *qnrS* (Kariuki and Onsare, 2015; Gómez-Baltazar et al., 2019; Monte et al., 2020; Yao et al., 2020; Xiaoting et al., 2021).

Our genomic Beast tree analysis provided evidence for the closer relationship among *bla*_CTX-M_-positive strains from the outpatients in this study and pork. Pig has been singled out as the most likely reservoir for the amplification and spread of Enterobacteriaceae that are resistant to ESBL and other antibiotics (Nordmann and Poirel, 2016), The same major *bla*_CTX-M_, as presented in this study, was also detected in isolates from a pig farm in China (Zhang et al., 2019). Therefore, our study provides strong genome epidemiology-based evidence that the consumption of pork is the likely contamination source of *bla*_CTX-M_-positive *S.* Typhimurium.

In the current study, most *bla*_CTX-M-55/14/65_ genes identified were carried by IncI1, IncHI2, and IncFII plasmids, which indicated that plasmids belonged to *bla*_CTX-M-55/14/65_-positive isolates and were diverse. Among them, IncI1 has become one of the most common plasmid families in contemporary Enterobacteriaceae from both human and animal sources. In clinical epidemiology, IncI1 ranks first as the confirmed vehicle of the transmission of extended spectrum beta-lactamase and *AmpC* genes in isolates from food-producing animals (Carattoli et al., 2021). The second, HI2, followed by FII plasmid, was found to be associated with the transfer of the *mcr-1* and ESBL encoding genes all over the world, especially in European and African countries. The coexistence of *mcr-1* and ESBL encoding genes in HI2 plasmids was less reported in China in recent years (Biswas et al., 2019; Wang et al., 2020). Worryingly, as the vector of drug resistance gene, FII plasmid not only carries *mcr-1*, but also is one of the common carriers of NDM gene (Wu et al., 2019).

WGS analysis further revealed that *bla*_CTX-M-55/14/65_ coexisted with other 25 types of ARGs, of which 11 ARGs were highly prevalent with detection rates >50%. Of note, *mcr-1*, conferring resistance to the last-resort antibiotic colistin, was detected in seven *bla*_CTX-M_-positive *S.* Typhimurium isolates. To begin with, the coexistence of *mcr-1* and *bla*_CTX-M-55_ was first reported in the literature from colistin-resistant clinical source *E. coli* isolates in Ecuador in 2016 (Ortega-Paredes et al., 2016). Next, the coexistence of *mcr-1* and ESBL encoding genes (including *bla*_CTX-M-55/14_) has been found in Tunisian from chicken, in China from food animal (including pigs, cattle and chickens) and in France from human *E. coli* (Birgy et al., 2018; Hassen et al., 2020; Shafiq et al., 2021). The coexistence of *mcr-1* and *bla*_CTX-M_ in *Salmonella* isolates was mostly reported from food animal sources and found in Asian countries, including China, Cambodia and Laos (Ma et al., 2017; Lay et al., 2021). Last but not least, the coexistence of *mcr-1*, *bla*_NDM-5_, and *bla*_CTX-M-55_ in *Klebsiella pneumoniae* ST485 Clinical Isolates appeared in China (Cao X. et al., 2021), which further alerted us to the dangers of multidrug-resistant strains.

## Conclusion

In summary, our study investigated the epidemiology of *S.* Typhimurium in Guangdong province, China. which could supplement important local epidemiological data. Among them, ST34 *S.* Typhimurium dominated the cefotaxime-resistant strains and the major resistance mechanism of cefotaxime-resistant *Salmonella* produced the CTX-M-type ESBLs, in which *bla*_CTX-M-55_ was most prevalent. Obviously, the prevalence of *bla*_CTX-M_-positive *S.* Typhimurium carried multiple resistance genes, which indicated the potential risk of *Salmonella* infections. In the current study, *bla*_CTX-M-55/65_-positive *S.* Typhimurium isolates were found from different outpatients with community acquired diarrhoea at same hospital, which suggested the nosocomial cloning transmission. This study underscored the importance of surveillance for *bla*_CTX-M_-positive microbes in patients and indicated a high likelihood for the spread of cephalosporin resistance from pig chain to humans.

## Data Availability Statement

The datasets presented in this study can be found in online repositories. The names of the repository/repositories and accession number(s) can be found in the article/Supplementary Material.

## Ethics Statement

Ethical review and approval was not required for the study on human participants in accordance with the local legislation and institutional requirements. Written informed consent for participation was not required for this study in accordance with the national legislation and the institutional requirements.

## Author Contributions

QJ wrote the first draft of the manuscript. QJ and L-xF contributed to conception and design of the study. B-xK, D-sW, DW, M-gW, R-yS, J-eL, and ZS performed the statistical analysis. All authors contributed to manuscript revision, read, and approved the submitted version.

## Funding

This work was jointly supported by the International Cooperation and Exchange of the National Natural Science Foundation of China (grant no. 31520103918), the Program for Changjiang Scholars and Innovative Research Team in University of Ministry of Education of China IRT_17R39, the National Natural Science Fund of China (grant no. 31802244), and the National Science and Technology Major Project (2018ZX10714002).

## Conflict of Interest

The authors declare that the research was conducted in the absence of any commercial or financial relationships that could be construed as a potential conflict of interest.

## Publisher’s Note

All claims expressed in this article are solely those of the authors and do not necessarily represent those of their affiliated organizations, or those of the publisher, the editors and the reviewers. Any product that may be evaluated in this article, or claim that may be made by its manufacturer, is not guaranteed or endorsed by the publisher.

## Footnotes

^1^ https://cge.cbs.dtu.dk/services/

## Supplementary Material

Supplementary Figure S1 Genetic relatedness, year, city and hospital of the *bla*_CTX-M_-positive *Salmonella* Typhimurium isolates in Guangdong from 2010 to 2017. **(A,B)** Genetic relatedness, year, city, hospital and pulsotype of the *bla*_CTX-M-55_-positive *S*. Typhimurium isolates **(C)** genetic relatedness, year, city, hospital and pulsotype of the *bla*_CTX-M-14_-positive *S*. Typhimurium isolates **(D)** genetic relatedness, year, city, hospital and pulsotype of the *bla*_CTX-M-65_-positive *S*. Typhimurium isolates.

Supplementary Figure S2 Genetic relatedness, year, city and hospital of the *bla*_CTX-M-55_-positive *S*. Typhimurium isolates in Guangdong from 2010 to 2017.

Supplementary Figure S3 Genetic relatedness, year, city and hospital of the *bla*_CTX-M-14_-positive *S*. Typhimurium isolates in Guangdong from 2010 to 2017.

Supplementary Figure S4 Genetic relatedness, year, city and hospital of the *bla*_CTX-M-65_-positive *S*. Typhimurium isolates in Guangdong from 2010 to 2017.

Supplementary Figure S5 S1 endonuclease pulsed-field gel electrophoresis analysis of plasmids from the *bla*_CTX-M-1G_-positive *S*. Typhimurium isolates.

Supplementary Figure S6 S1 endonuclease pulsed-field gel electrophoresis analysis of plasmids from the *bla*_CTX-M-9G_-positive *S*. Typhimurium isolates.

Supplementary Figure S7 PBRT types that *bla*_CTX-M_-positive *S*. Typhimurium isolates **(A)** all PBRT types that *bla*_CTX-M_-positive *S*. Typhimurium isolates **(B)** PBRT types that *bla*_CTX-M-55_-positive *S*. Typhimurium isolates. **(C)** PBRT types that *bla*_CTX-M-14_-positive *S*. Typhimurium isolates. **(D)** PBRT types that *bla*_CTX-M-65_-positive *S*. Typhimurium isolates.
