# Supplementary figures and images for: Epidemiology of blaCTX-M-Positive Salmonella Typhimurium From Diarrhoeal Outpatients in Guangdong, China, 2010–2017
Source: Front Microbiol. 2022 Jun 17;13:865254. doi: 10.3389/fmicb.2022.865254 (PMC9247517; doi:10.3389/fmicb.2022.865254)

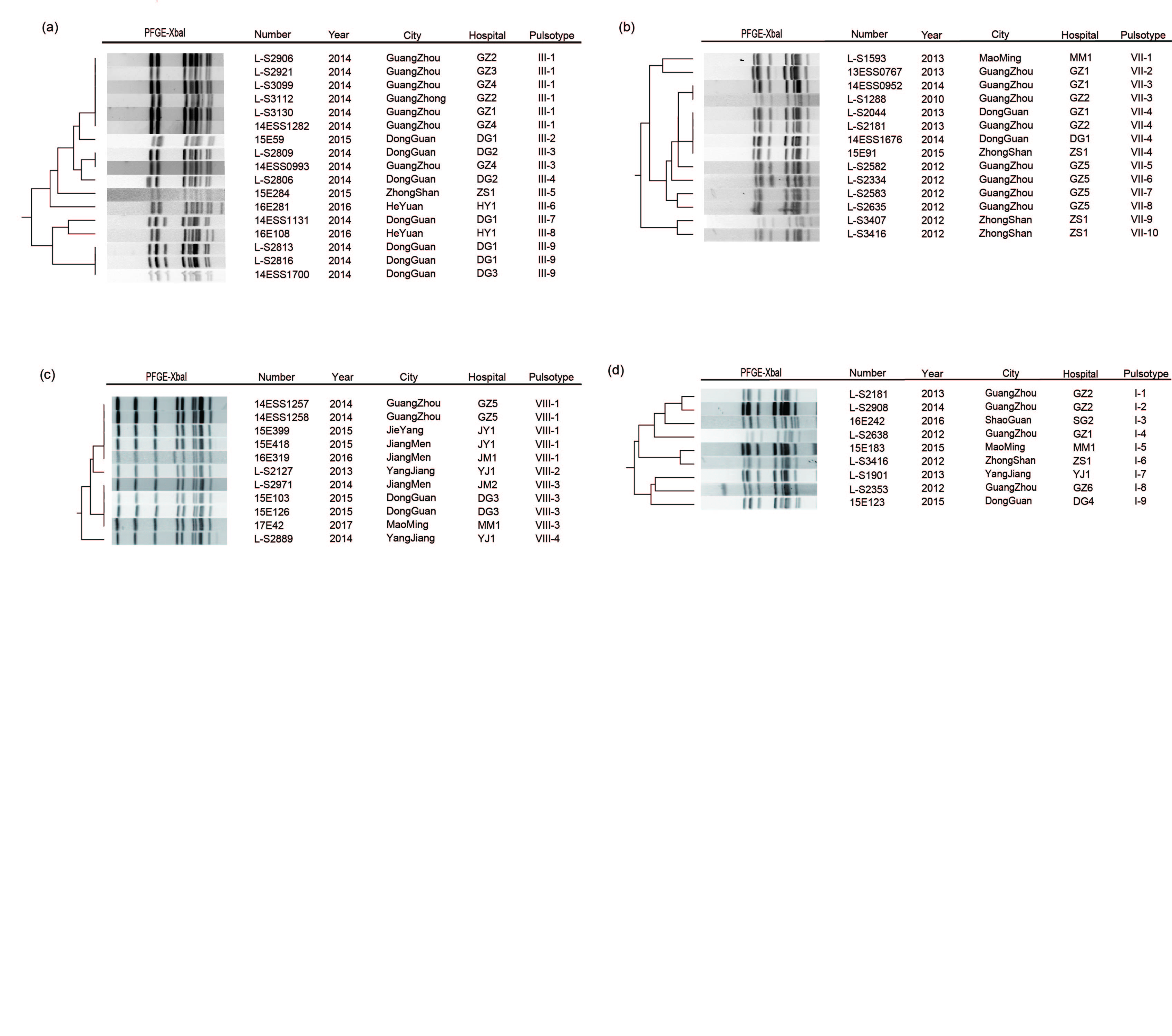

Supplement: Supplementary Figure S1 — Genetic relatedness, year, city and hospital of the blaCTX-M-positive Salmonella Typhimurium isolates in Guangdong from 2010 to 2017. (A,B) Genetic relatedness, year, city, hospital and pulsotype of the blaCTX-M-55-positive S. Typhimurium isolates (C) genetic relatedness, year, city, hospital and pulsotype of the blaCTX-M-14-positive S. Typhimurium isolates (D) genetic relatedness, year, city, hospital and pulsotype of the blaCTX-M-65-positive S. Typhimurium isolates. [file Image_1.JPEG]

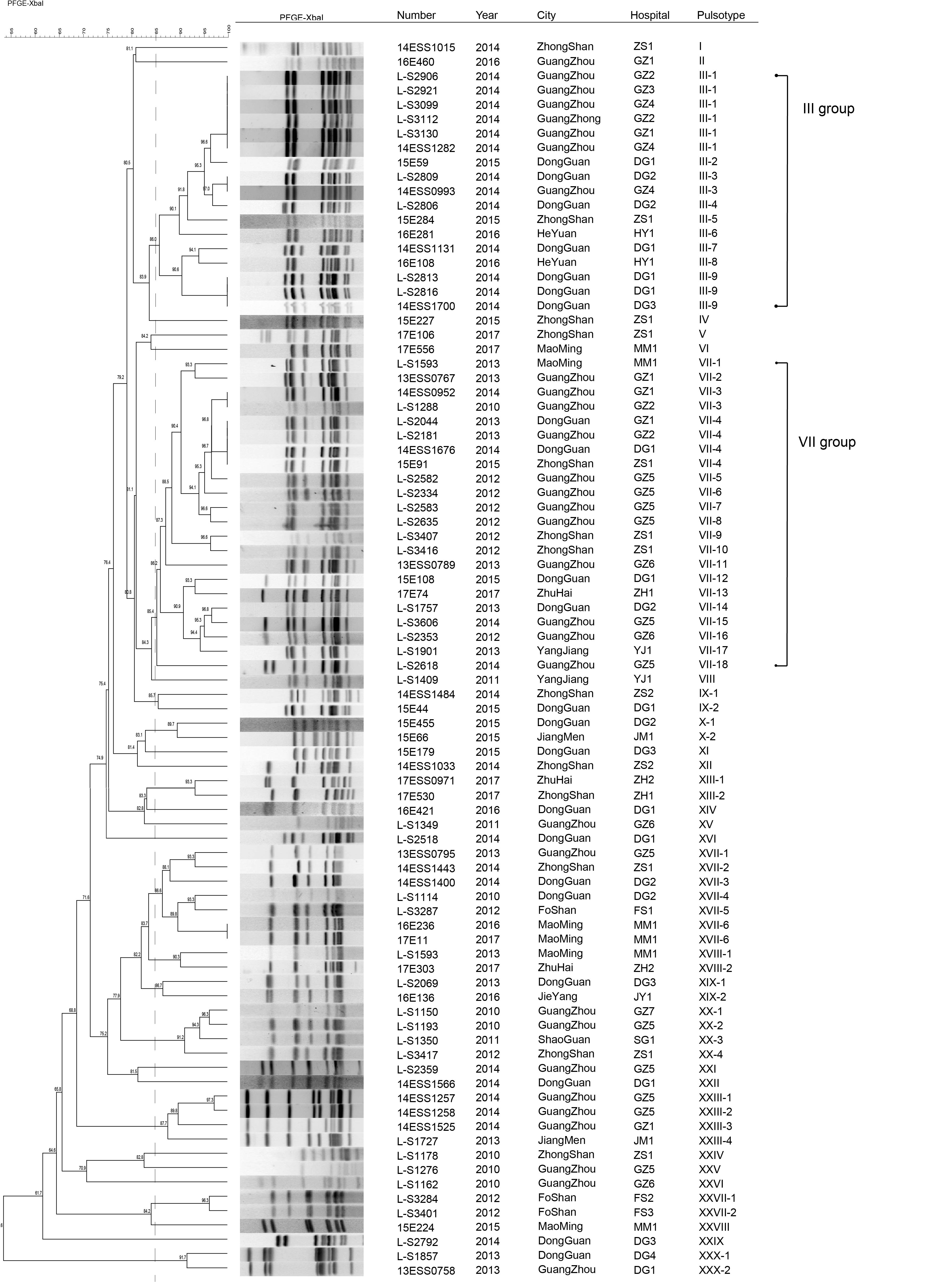

Supplement: Supplementary Figure S2 — Genetic relatedness, year, city and hospital of the blaCTX-M-55-positive S. Typhimurium isolates in Guangdong from 2010 to 2017. [file Image_2.JPEG]

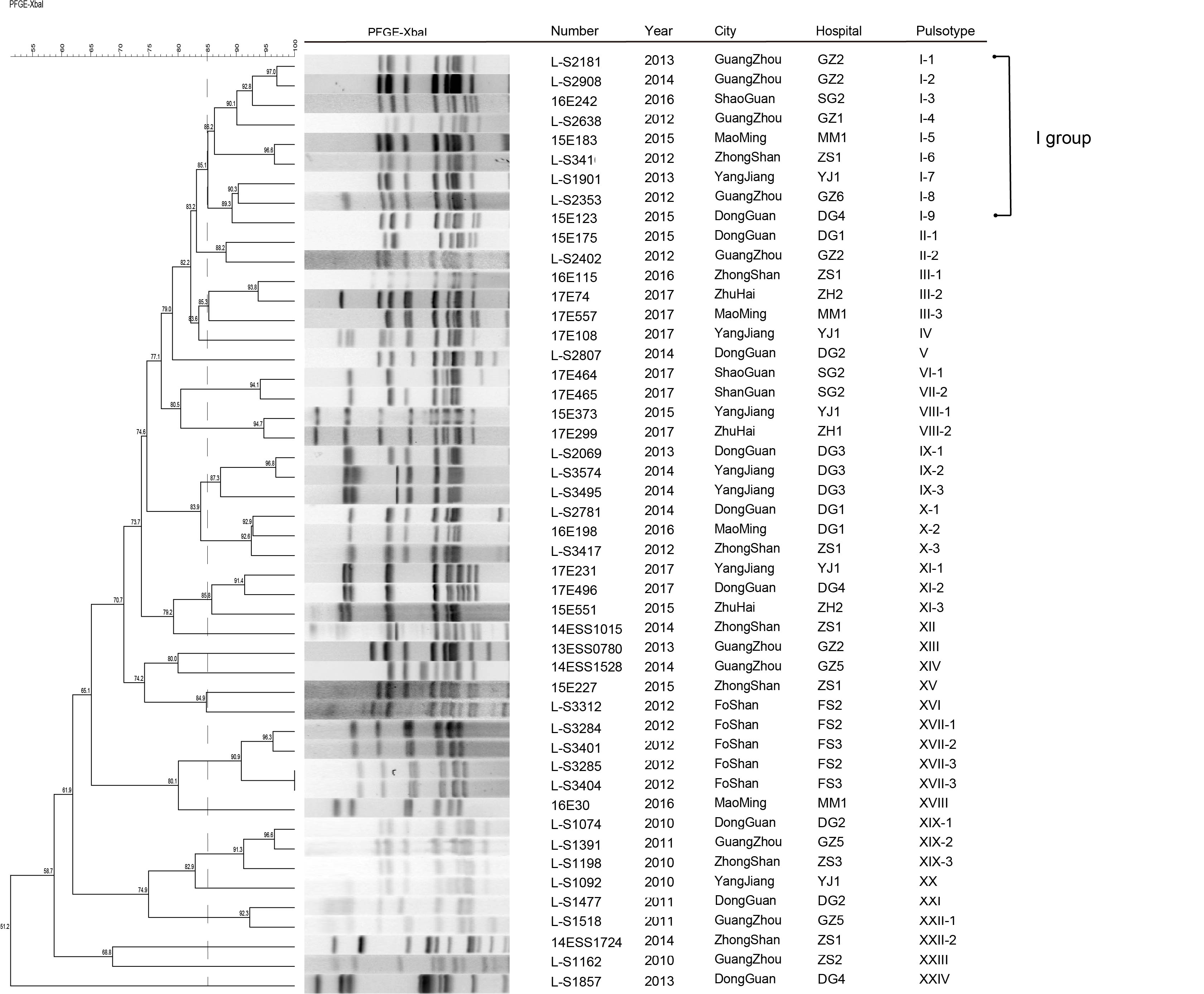

Supplement: Supplementary Figure S3 — Genetic relatedness, year, city and hospital of the blaCTX-M-14-positive S. Typhimurium isolates in Guangdong from 2010 to 2017. [file Image_3.JPEG]

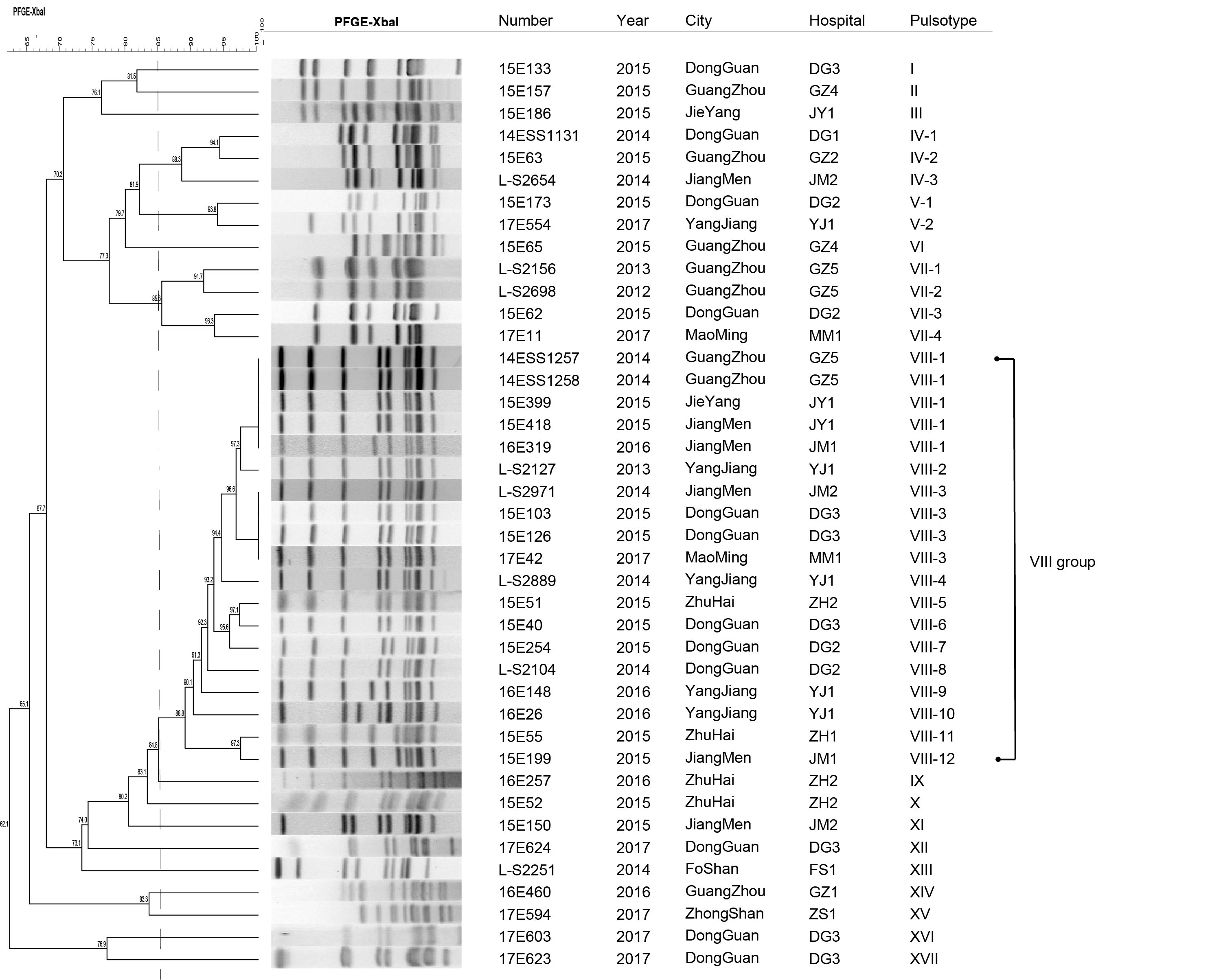

Supplement: Supplementary Figure S4 — Genetic relatedness, year, city and hospital of the blaCTX-M-65-positive S. Typhimurium isolates in Guangdong from 2010 to 2017. [file Image_4.JPEG]

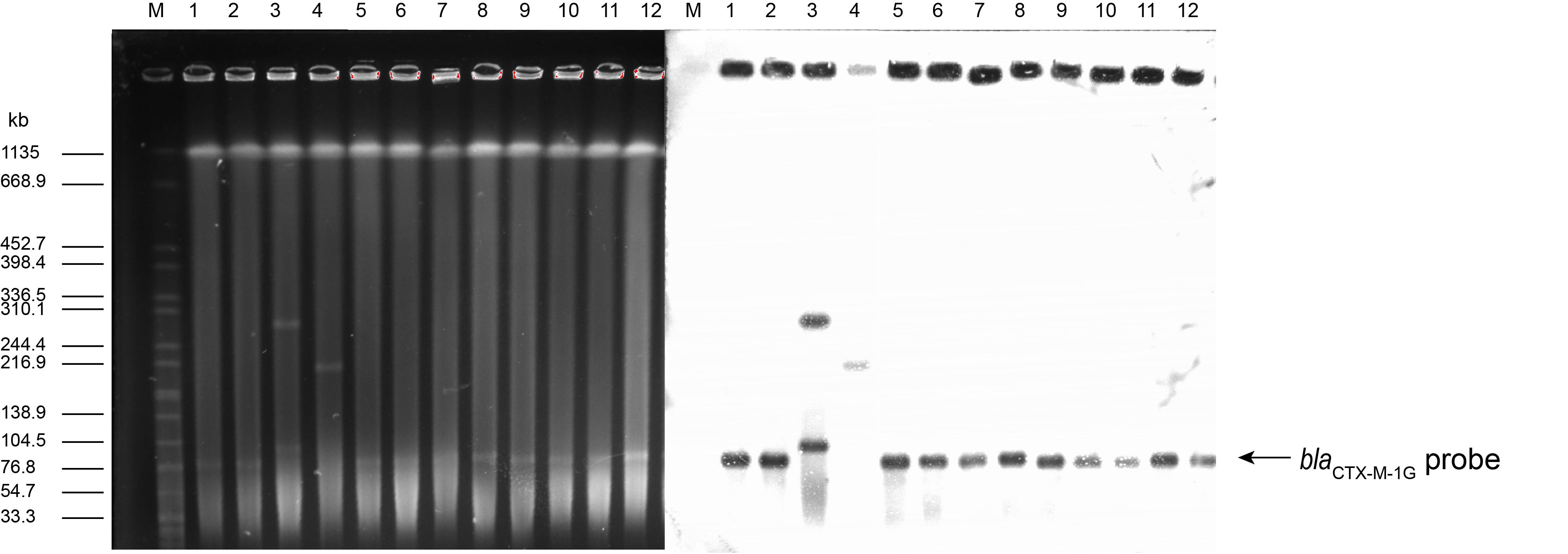

Supplement: Supplementary Figure S5 — S1 endonuclease pulsed-field gel electrophoresis analysis of plasmids from the blaCTX-M-1G-positive S. Typhimurium isolates. [file Image_5.JPEG]

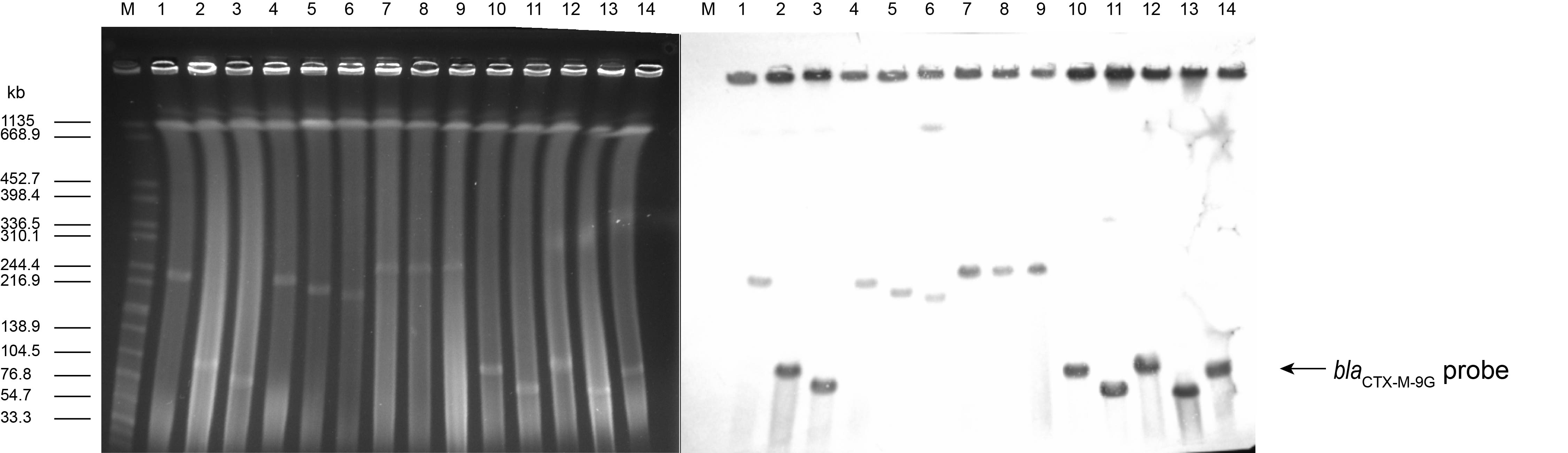

Supplement: Supplementary Figure S6 — S1 endonuclease pulsed-field gel electrophoresis analysis of plasmids from the blaCTX-M-9G-positive S. Typhimurium isolates. [file Image_6.JPEG]

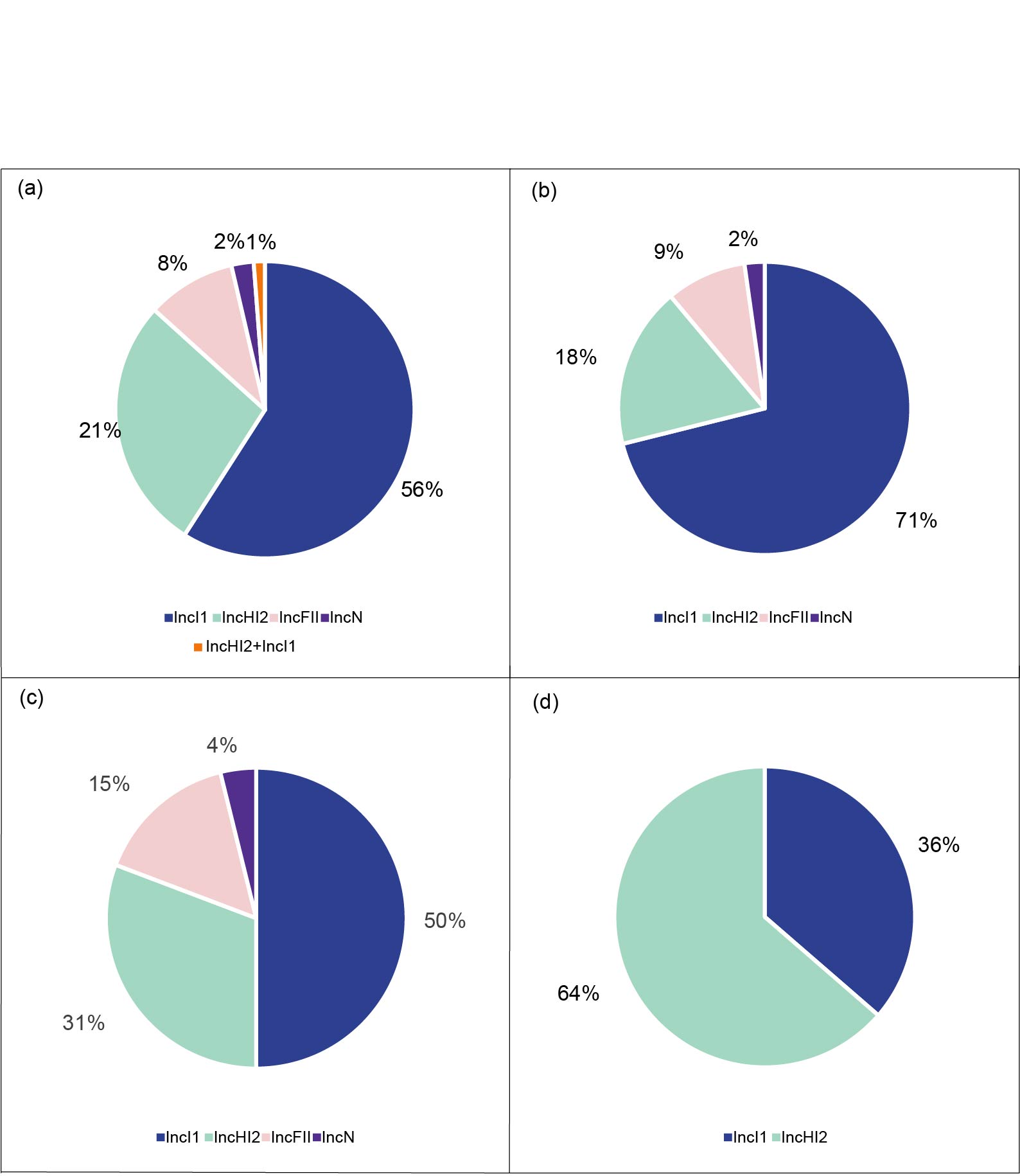

Supplement: Supplementary Figure S7 — PBRT types that blaCTX-M-positive S. Typhimurium isolates (A) all PBRT types that blaCTX-M-positive S. Typhimurium isolates (B) PBRT types that blaCTX-M-55-positive S. Typhimurium isolates. (C) PBRT types that blaCTX-M-14-positive S. Typhimurium isolates. (D) PBRT types that blaCTX-M-65-positive S. Typhimurium isolates. [file Image_7.JPEG]
