# Supplementary material for: Epidemiology of blaCTX-M-Positive Salmonella Typhimurium From Diarrhoeal Outpatients in Guangdong, China, 2010–2017
Source: Front Microbiol. 2022 Jun 17;13:865254. doi: 10.3389/fmicb.2022.865254 (PMC9247517; doi:10.3389/fmicb.2022.865254)
Supplement: Supplementary file 8 [file Table_1.docx]

**Table S1**. Primers used for amplification.

| **Primer** | **Sequence（5’-3’）** | **Reference** |
| --- | --- | --- |
| *bla*_CTX-M_-F | TTTGCGATGTGCAGTACCAG | (Liu et al, 2013) |
| *bla*_CTX-M_-R | CGATATCGTTGGTGGTGCC |  |
| 1G.F | CGCTTTGCGATGTGCAG | （Paterson et al, 2003） |
| 1G.R | ACCGCGATATCGTTGGT |  |
| 9G.F | GCAGTACAGCGACAATACCG | （Grobner et al, 2009） |
| 9G.R | TATCATTGGTGGTGCCGTAG |  |
| *bla*_SHV_-F | TTCGCCTGTGTATTATCTCC | （Grobner et al, 2009） |
| *bla*_SHV_-R | TCCGCTCTGCTTTGTTATTC |  |
| *bla*_CMY-2G_-F | GCACTTAGCCACCTATACGGCAG | (Liu et al, 2007) |
| *bla*_CMY-2G_-R | GCTTTTCAAGAATGCGCCAGG |  |
| *bla*_DHA_-F | TGATGGCACAGCAGGATATTC | (Liu et al, 2007) |
| *bla*_DHA_-R | GCTTTGACTCTTTCGGTATTCG |  |
| ISEcp1.F | CTATCCGTACAAGGGAGTGT | （Sun et al, 2009） |
| orf477 | CAGCGGAAGGAGAACCAG | （Sun et al, 2009） |
| IS903 | TTTCCACTCGCCTTCACC | （Sun et al, 2009） |
